# Evaluation of a radiomics workflow in chest CT: a pilot study of lung lesion segmentation, feature extraction, and clustering

**DOI:** 10.1590/0100-3984.2025.0005

**Published:** 2026-09-08

**Authors:** Ana Carolina Costa da Silva, Paulo Henrique Ruis Garcia, Karina Yukimi Pexito Sakurai, Douglas Carli Silva, Maria Vitória David Ludwig, Fabiana Reis Decicino Campos, Márcio Valente Yamada Sawamura, Hye Ju Lee

**Affiliations:** 1 Siemens Healthineers, Computed Tomography, São Paulo, SP, Brazil; 2 Institute of Energy and Nuclear Research, University of São Paulo, São Paulo, SP, Brazil; 3 Siemens Healthineers, Education Services, São Paulo, SP, Brazil; 4 Institute of Radiology, Hospital das Clínicas, University of São Paulo School of Medicine, São Paulo, SP, Brazil; 5 Hospital Sírio-Libanês, São Paulo, SP, Brazil

**Keywords:** Radiomics, Lung neoplasms, Tomography, X-ray computed, Radiographic image interpretation, computer-assisted, Cluster analysis, Biomarkers, Machine learning., Radiômica, Neoplasias pulmonares, Tomografia computadorizada por raios X, Interpretação de imagem radiográfica assistida por computador, Análise por conglomerados, Biomarcadores, Aprendizado de máquina

## Abstract

**Objective:**

To evaluate a radiomics workflow applied to chest computed tomography (CT), examining its feasibility and performance across the steps of lung lesion segmentation, feature extraction, and clustering, as well as exploring its potential to distinguish between benign and malignant lung lesions.

**Materials and Methods:**

This was a retrospective study including CT images of 32 histopathology-confirmed lesions (11 benign; 21 malignant). Lesions were segmented by using syngo.via Frontier Radiomics software, af-ter which > 900 radiomic features were extracted with the same software. Data processing-including normal-ization, dimensionality reduction (principal component analysis), and unsupervised (k-means) clustering-was conducted in Python.

**Results:**

The best clustering solution was obtained with a k = 2 (silhouette score = 0.4668), effectively separating benign lesions, which exhibited low heterogeneity, from malignant lesions, which exhibited complex patterns of texture and shape.

**Conclusion:**

The application of a radiomics workflow proved feasible and potentially useful for differentiating between benign and malignant lung lesions on CT, underscoring its value as a complementary tool in thoracic oncology. How-ever, future studies using multicenter datasets, blinded evaluations, and more structured integration environments are warranted in order to support clinical validation and prototyping.

## Abstract (pt)

**Objetivo:**

Avaliar um fluxo de trabalho em radiômica aplicado à tomografia computadorizada de tórax, examinando a viabilidade e desempenho nas etapas de segmentação de lesões pulmonares, extração de características e agrupa-mento, além de explorar o potencial para distinguir lesões pulmonares benignas de malignas.

**Materiais e Métodos:**

Estudo retrospectivo incluindo 32 lesões pulmonares com diagnóstico histopatológico (11 be-nignas, 21 malignas). As segmentações das lesões foram realizadas com o syngo.via MM Radiomics Frontier, seguido da extração de > 900 características radiômicas pelo mesmo software. Os dados foram processados em Python com normalização, redução de dimensionalidade (PCA) e agrupamento não supervisionado (k-means).

**Resultados:**

O modelo k-means com k = 2 apresentou melhor desempenho (coeficiente de silhueta = 0,4668), distin-guindo adequadamente lesões benignas, com baixa heterogeneidade, das malignas, caracterizadas por maior com-plexidade textural e morfológica.

**Conclusão:**

A aplicação da radiômica mostrou-se factível e potencialmente útil na diferenciação entre lesões pulmo-nares benignas e malignas em exames de TC, reforçando seu valor como ferramenta complementar em oncologia torácica. No entanto, recomenda-se a realização de estudos futuros com conjuntos de dados multicêntricos, avalia-ções cegas e ambientes de integração mais estruturados para validação e prototipagem clínica.

## INTRODUCTION

Lung cancer remains one of the leading causes of cancer-related mortality worldwide, accounting for approximately 1.8 million deaths annually^**(^1^)**^. Early de-tection is critical to improving survival rates, and chest computed tomography (CT) plays a pivotal role in lung cancer screening and diagnosis, particularly among high-risk populations^**(^2^)**^.

Radiomics has emerged as a powerful approach that extracts quantitative features from standard medical im-ages. These features, derived from pixel intensity dis-tributions and spatial relationships, can capture subtle textural, morphological, and structural properties of tis-sue, which may correlate with histological and genetic tumor characteristics^**(^3^,^4^)**^. Radiomic features are typically categorized as first-order statistics (e.g., histogram-based features), second-order texture metrics (e.g., gray level co-occurrence matrix [GLCM] and gray level run-length matrix [GLRLM]), and higher-order features, which in-corporate transformations such as wavelets or filters to capture multiscale patterns.

The central premise of radiomics is that medical images contain hidden biological information not always apparent in visual interpretation. By combining radiomic data with clinical or molecular information, it is possible to generate predictive models that may support clinical decision-making, as well as facilitating diagnosis, prog-nostication, and treatment planning^**(^5^,^6^)**^.

In the context of lung cancer, chest CT is particularly well-suited for radiomic analysis, as it allows detailed visu-alization of pulmonary nodules and masses. However, the success of radiomics depends heavily on a standardized, reproducible workflow, including consistent acquisition parameters, robust segmentation, and careful feature selection. Variability in any of these steps can compromise the stability and generalizability of radiomic models^**(^7^,^8^)**^.

The aim of this study was to present a pilot project that explores the best practices in radiomics applied to chest CT for the detection and classification of lung lesions, including benign, primary malignant, and secondary malignant (metastatic) lesions. This work highlights the importance of a structured workflow, interdisciplinary collaboration, and explainability in exploratory radiomics analyses.

## MATERIALS AND METHODS

This retrospective study was approved by the local institutional review board (Reference no. 78384324.5.0000.5461). Because the data were collected retrospectively, the requirement for informed consent was waived. Chest CT images were obtained from onco-logic patients under investigation for a new finding of a pulmonary nodule during follow-up CT. All lesions were submitted to CT-guided biopsy. A total of 32 pulmonary lesions were included in this pilot study, comprising 11 benign lesions and 21 malignant lesions. Malignant le-sions were further classified as either primary lung tumors or metastatic lesions. No treatment or clinical outcome was considered for classification. The dataset, including diagnoses and subclassifications, is presented in Table 1.

**Table 1:** Dataset composition: histopathological diagnosis and subclassification of lung lesions.

Parameter	(N = 32)
Sex, n	
Male	12
Female	20
Age (years), mean (range)	65.7 (41-91)
Benign lesions,^*^ n	11
Primary malignant lesions,^†^ n	14
Secondary malignant lesions,^‡^ n	7

* Organizing necrotic tissue with chronic granulomatous inflammation, aspergillus, necrotic inflammatory nodule, or a chronic nodular inflammatory process with fibrosis and lymphoplasmacytic infiltrate.

† Lung adenocarcinoma or poorly differentiated non-small cell lung cancer.

‡ Metastasis from Hodgkin lymphoma, renal cancer, leiomyosarcoma, breast cancer, or melanoma.

Chest CT examinations were performed using multimodal scanners equipped with proprietary itera-tive reconstruction algorithms: Biograph 128 (Siemens Healthineers, Erlangen, Germany); Somatom Defini-tion AS+ (Siemens Healthineers); Somaton Definition AS (Siemens Healthineers); Somatom Definition Edge (Siemens Healthineers); and Discovery MI (GE Health-care, Milwaukee, WI, USA). Tube voltage and current modulation were adjusted according to vendor specific technology; in the sample as a whole, the mean value was 120 kVp (range, 90-140 kVp). All images were acquired in helical mode during a full-inspiration breath-hold, with the following parameters: slice thickness, 1.0-2.0 mm with matching reconstruction intervals; 512×512 matrix; and a field of view adjusted to the thoracic cav-ity. To minimize variability during lesion segmentation and radiomic feature extraction, all scans were acquired through the use of unenhanced chest CT protocols and the images were reconstructed using standard soft-tissue and lung kernels , thus allowing adequate evaluation of parenchymal texture and lesion morphology.

We segmented the lesions by using the software syngo. via MM Radiomics Frontier (Siemens Healthineers), em-ploying a semi-automated approach followed by manual refinement. All segmentations were performed by an experienced thoracic radiologist, to ensure anatomical accuracy and consistency. Only unenhanced axial im-age reconstructions were used for segmentation and radiomics analysis.

Following segmentation, a comprehensive set of > 900 radiomic features was extracted for each lesion using the syngo.via MM Radiomics Frontier software, which makes use of the PyRadiomics package and offers the option of feature computation/extraction, as well as al-lowing the results to be exported in the comma-separated values format ^**(^9^)**^. The extracted features encompassed first-order statistics, shape descriptors, and high-order texture metrics derived from matrices such as GLRLM, GLCM, gray level size-zone matrix, and wavelet trans-formations. Feature selection was also used to reduce not-a-number values in order to explore redundancy and correlation among radiomic features, we conducted a pairwise Pearson correlation analysis, visualized on a clustered heatmap. After feature selection, 26 variables remained and were used for the final analysis.

The analysis was performed using custom Python scripts implemented through the feature analysis mod-ule, with statistical packages for analytics. The analytical pipeline included the following steps:

Feature normalization was conducted with Standard-Scaler to standardize all features to have zero mean and unit variance, thus ensuring equal contribution during clustering.

Dimensionality reduction was performed via princi-pal component analysis (PCA) to reduce feature space complexity and allow two-dimensional visualization of cluster separation.

Unsupervised clustering was carried out by using the k-means algorithm, with the number of clusters (k) vary-ing from 2 to 5 to explore different grouping structures. Cluster validity was assessed by calculating the sil-houette score, a metric that quantifies how well each sample fits within its assigned cluster compared with other clusters. Higher scores (closer to 1) indicate clustering structures that are more cohesive and well-defined.

**Figure 1:**
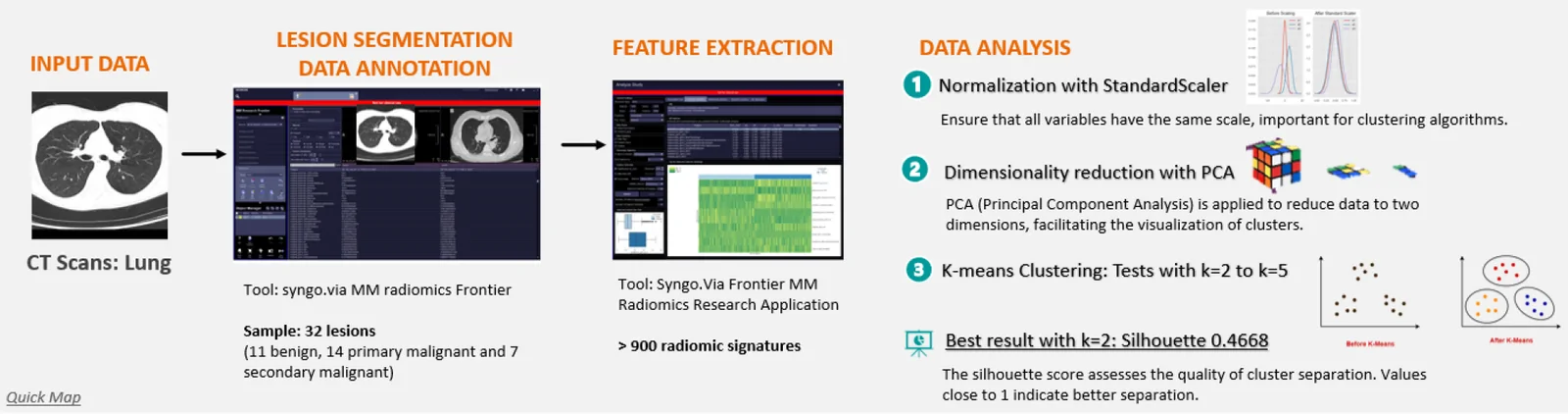
Quick map project, created by the authors.

**Figure 2:**
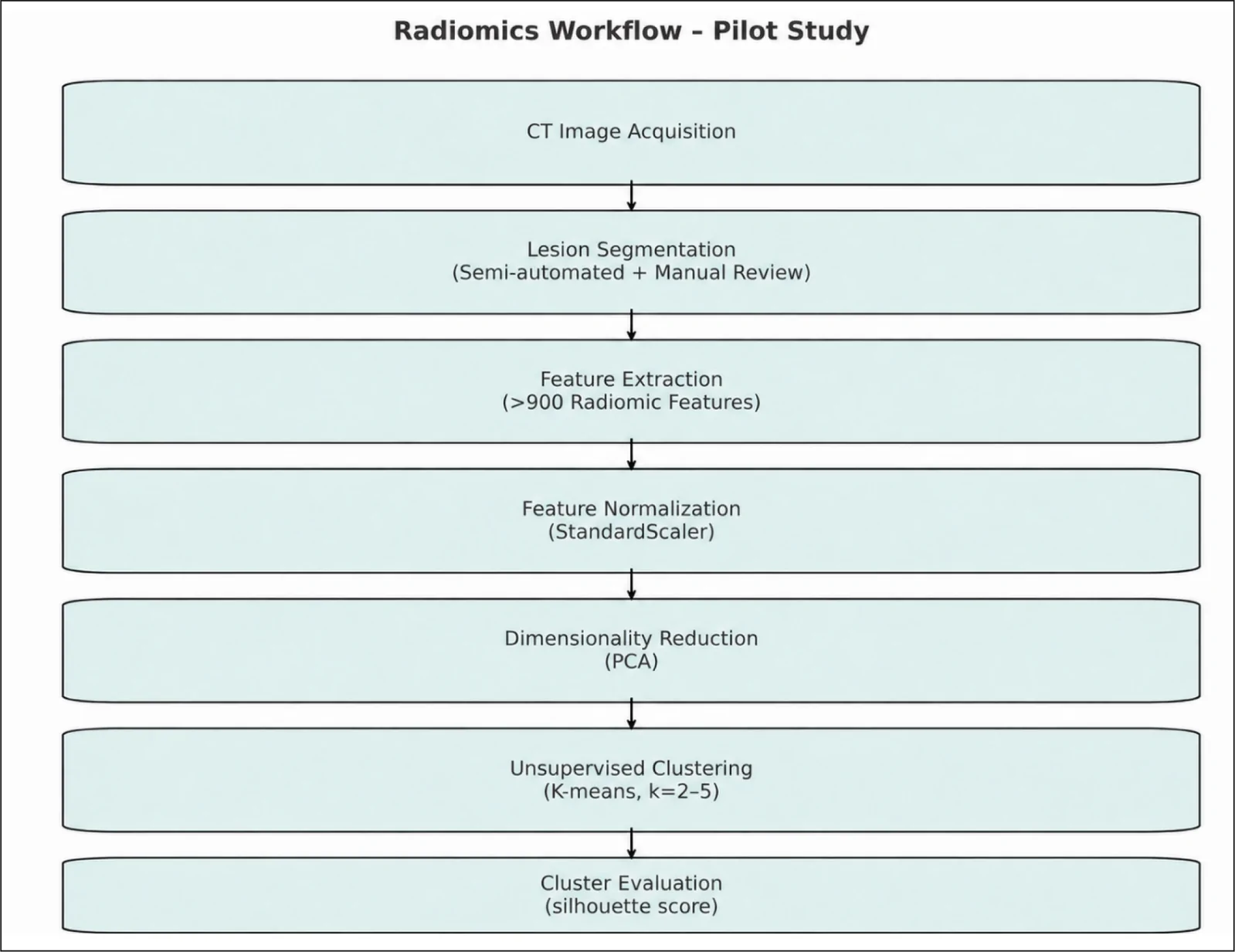
Workflow pilot study, created by the authors.

Supervised models with clustered data were tested to evaluate the feasibility of results and the potential of the study to validate the workflow using radiomics analysis. The Radiomics workflow is illustrated and summarized in the Figures 1 and 2.

To evaluate the classification performance of the models, four supervised machine learning algorithms were trained and tested: Random Forest, Logistic Re-gression, Support Vector Machine (SVM), and eXtreme Gradient Boosting (XGBoost). The receiver operating characteristic (ROC) curves were plotted with the predicted probabilities for the positive class, and the areas under the curve (AUCs) were used as the primary metric for model comparison. All models were evalu-ated on the same test dataset using the same feature set. This approach allows for a consistent assessment of the ability of each model to distinguish between classes, regardless of the decision thresholds.

## RESULTS

**Figure 3:**
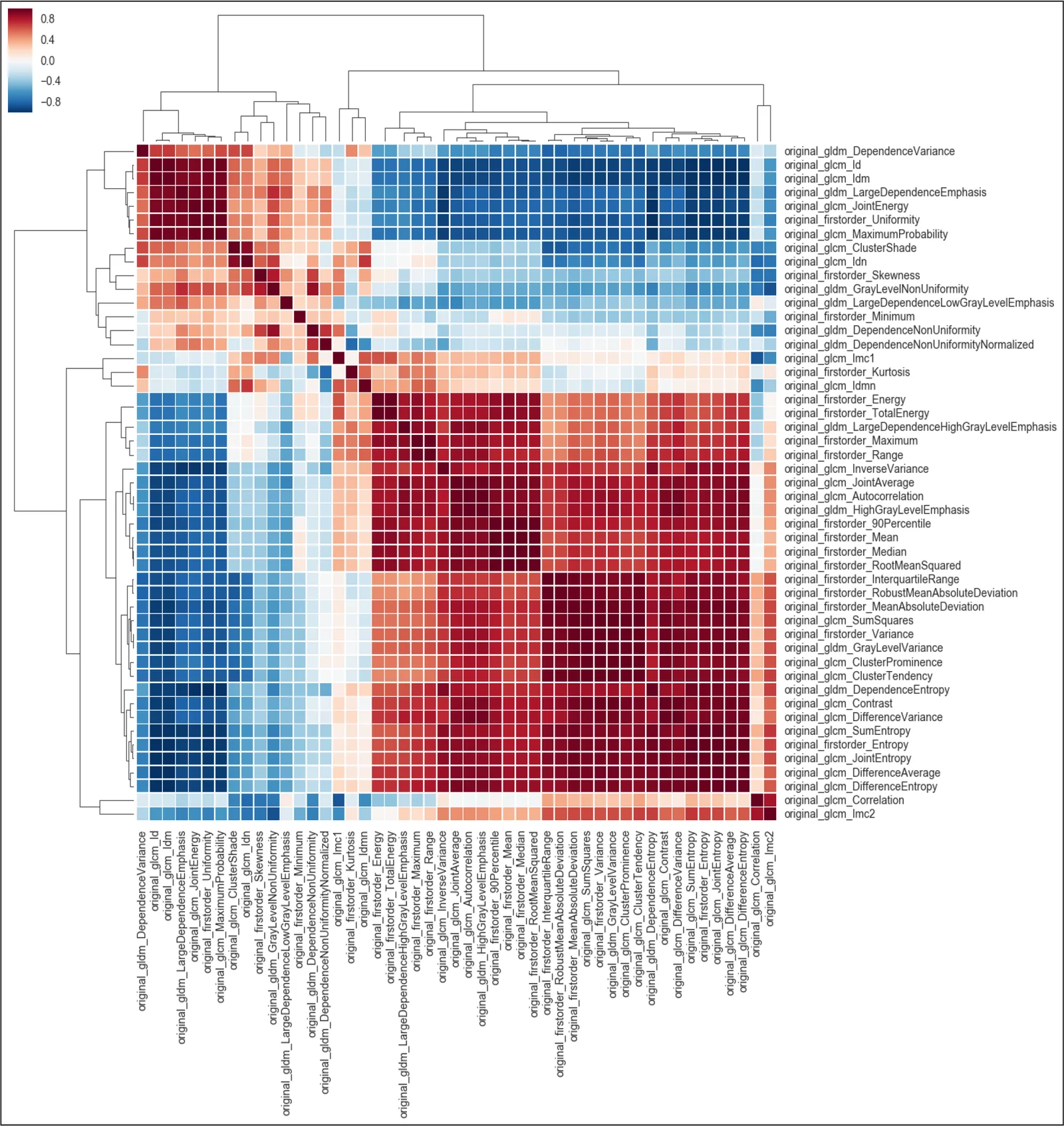
Clustered heatmap of pairwise Pearson correlation coefficients among radiomic features. Red indicates a strong positive cor-relation, and blue indicates a strong negative correlation. Hierarchical clustering was applied to group similar features, enabling the iden-tification of redundant or collinear variables for potential feature selection.

Radiomics extraction provides a large number of data, and feature selection is a crucial step in the radiomics workflow. The goal is to reduce irrelevant or redundant features and retain those that are relevant and useful, reducing dimensionality so that analysis process can be performed more efficiently, decreasing the resource demand, work time, and risk of overfitting^**(^10^)**^. To achieve that reduction, we conducted a pairwise Pearson cor-relation analysis to explore redundancy and correlation among radiomic features, visualized through a clustered heatmap (Figure 3). The matrix displays the correlation coefficients between features. Hierarchical clustering was applied to groups of highly correlated features, helping to identify patterns and potential collinearities in the dataset. This step is crucial for feature selection, because highly correlated variables may contribute redundant information to downstream models and affect their generalizability.

In the differentiation between benign and malig-nant lesions, the analysis highlighted several feature clusters with strong internal correlations, suggesting opportunities for dimensionality reduction or selection of representative features within each cluster.

After this first feature selection, k-means analysis was applied to data to classify into groups. Among the tested configurations, the clustering solution with k = 2 produced the highest silhouette score (0.4668), indicat-ing the most distinct and internally coherent separation of data points. This suggests that the dataset naturally tends to form two well-defined clusters. In contrast, lower silhouette scores were observed for other cluster configurations: k = 3 (0.2763), k = 4 (0.2889), and k = 5 (0.3860), reflecting less cohesive groupings and overlap-ping between clusters. The PCA projections supported those findings, showing visually interpretable cluster separation when k = 2, which appeared to align with the binary classification of lesions as benign or malignant.

In the subgroup analysis of malignant lesions, no statistically significant differences were observed between primary and metastatic malignant tumors. The unsuper-vised k-means clustering with k = 2, which yielded the highest silhouette score (0.4668), grouped primary and secondary malignant lesions predominantly within the same cluster, indicating a high degree of similarity be-tween these subtypes. Consistently, the PCA projections demonstrated substantial overlap between primary and metastatic malignant lesions, with no clear separation into distinct clusters. These findings are further supported by the moderate performance of the supervised classification models, with the best model (XGBoost) achieving an AUC of 0.72, suggesting limited discrimi-natory ability between malignant subtypes within the constraints of the current feature set and the small sample size (n = 32).

**Figure 4:**
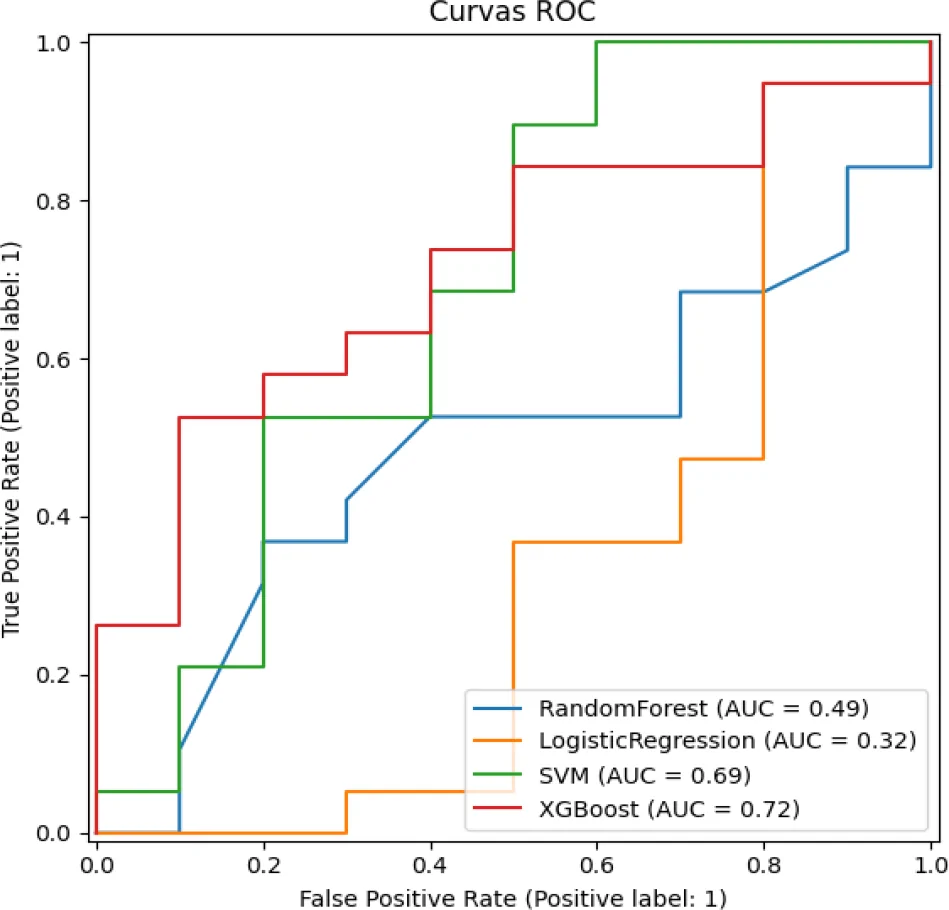
ROC curves for the four classification models tested. The AUC is shown in parentheses for each model. The XGBoost model outperformed the others, achieving an AUC of 0.72, fol-lowed by the SVM model (AUC = 0.69), whereas the Random For-est and Logistic Regression models performed near or below ran-dom classification levels.

The ROC curve analysis (Figure 4) compares the performance of four classification models: Random Forest, Logistic Regression, SVM, and XGBoost. Among them, the XGBoost model achieved the best performance, with an AUC of 0.72, followed by the SVM model, with an AUC of 0.69. In contrast, the Random Forest and Logistic Regression models demonstrated poor discriminatory power, with AUCs of 0.49 and 0.32, respectively, both below the level of chance (AUC = 0.5).

## Preliminary clinical interpretation of clusters

The two clusters identified through unsupervised k-means clustering (k = 2) demonstrated a clinically mean-ingful pattern when compared to the biopsy-confirmed classification of lesions.

Cluster 1 was predominantly composed of benign lesions, typically presenting more homogeneous texture, smoother borders, and lower intensity variation, as cap-tured by lower first-order entropy and less complex texture metrics (e.g., GLCM contrast and GLRLM run entropy).

Cluster 2 was composed mainly of malignant le-sions, including primary and secondary tumors. These lesions exhibited higher heterogeneity, irregular shapes, and greater variation in pixel intensity and texture, consistent with biological aggressiveness and struc-tural disorganization, characteristics well reflected in higher-order radiomic features.

## DISCUSSION

Our results suggest that radiomics features alone may be sufficient to distinguish between benign and malignant pulmonary lesions, even in the absence of clini-cal or molecular data. However, the cluster containing malignant lesions included primary and secondary (meta-static) tumors, which were not consistently separated by the current feature set or clustering method, suggesting that additional stratification strategies may be necessary to resolve finer subclass differences in future analyses.

From a clinical perspective, our findings indicate that radiomics could play a role as a decision-support tool for early triage of lung lesions detected on CT scans, helping to flag cases that require further diagnostic work-up. Nevertheless, the inability to distinguish between different malignant subtypes at this stage underscores the need for the following^**(^9^)**^:

Integration of clinical metadata, such as patient medical history/smoking status and overall disease severity (e.g., tumor-node-metastasis staging)

Inclusion of molecular markers (tumor histology)

Refinement of feature selection strategies focused on tumor biology

This pilot study demonstrated the practical feasibil-ity of implementing a structured radiomics workflow using chest CT data within a clinical setting. Beyond technical validation, it also underscored critical elements that influence the robustness and reproducibility of radiomics analyses, particularly the need for standard-ized acquisition parameters and consistent segmenta-tion practices. These findings are aligned with data in the current literature, supporting the idea that even subtle variations in CT protocol (e.g., slice thickness, reconstruction filters) can have a significant impact on feature stability and model performance^**(^11^,^12^)**^.

The clustering results when k = 2 was applied revealed that the extracted features captured relevant differences between benign and malignant lesions. This supports the underlying premise of radiomics: that imaging contains quantifiable patterns reflecting biological behavior, which may not be apparent through visual inspection alone. The concordance between the clustering structure and the ground truth classification suggests that radiomics descriptors hold significant discriminatory power and may serve as the foundation for future decision-support tools.

Dimensionality reduction methods such as PCA are essential for selecting a specific number of features for analysis, because they help identify redundant or highly correlated variables that contribute similarly to the out-come. These methods also facilitate data processing by reducing the computational resources required. However, a key drawback is the potential loss of model explainability, which may be a significant barrier to clinical adoption.

Several key challenges were identified throughout the project:

Workflow-Although semi-automated segmentation tools were used for this study, a manual review, which is time-intensive and limits scalability, is still required for the ultimate accuracy of segmentation. This highlights the importance of developing or integrating an automated segmentation tool validated for clinical use^**(^13^,^16^)**^.

Protocol harmonization-To ensure reproducibility, adherence to standardized acquisition parameters is essential. Inconsistent voxel sizes, slice thicknesses, or reconstruction kernels introduce variability that can obscure biologically relevant patterns. A resampling tool can be used to ensure isotropic voxels with equal distances between neighboring voxels in all directions. This step is crucial to ensure that the data extracted from the images is equivalent across the different types of studies analyzed^**(^12^-^14^)**^.

Multidisciplinary collaboration-Effective radiomics research demands the integration of expertise from radi-ology, data science, and imaging physics. This interdis-ciplinary effort was crucial for designing the analytical pipeline, selecting appropriate features, and interpreting the results in a clinically meaningful context^**(^15^)**^.

The dataset used in this pilot study comprised only 32 cases, which precluded a statistically reliable division into independent training and testing subsets. Consid-ering the limited sample size and the exploratory aim of the study, we employed an unsupervised clustering approach. This method does not require predefined training or validation partitions, given that the objective was not predictive modeling but the identification of intrinsic data patterns. Despite these constraints, the findings demonstrate the potential of radiomics features to discriminate between benign and malignant lung le-sions. In addition, this pilot study contributed to ongoing discussions on best practices for CT-based radiomics, particularly in the field of thoracic oncology^**(^16^,^17^)**^.

Key methodological pillars include the adoption of standardized image acquisition and reconstruction pro-tocols, ensuring image harmonization across different vendors. In addition, the careful selection of appropriate techniques for feature selection and dimensionality reduc-tion is critical to maintain a balance between efficient data processing and model interpretability^**(^16^)**^. Overall, these insights serve as a foundation for expanding this work into larger, multicentric studies. They also emphasize the value of radiomics not only as a research tool but as a potential enabler of precision imaging and data-driven decision-making in routine clinical care.

Finally, enriching the analysis with clinical variables like age, sex, and medical history may provide a more comprehensive model that better reflects real-world di-agnostic decision-making.

## CONCLUSION

This pilot study validated the feasibility of deploy-ing a radiomics pipeline in the clinical CT environment, specifically for the analysis of lung lesions. Through semi-automated segmentation feature extraction and data analysis, we demonstrated that radiomics descriptors can effectively differentiate benign from malignant lesions using unsupervised clustering techniques. The results underscore the potential of radiomics as a noninvasive, quantitative tool to support clinical decision-making, particularly in the early detection and characterization of lung malignancies. However, the study also highlighted key operational challenges, including the need for work-flow automation, strict protocol standardization, and integration of multidisciplinary expertise, that must be addressed to ensure scalability and reproducibility.

Future research should prioritize multicenter stud-ies to expand the dataset and ensure broader population diversity, thereby enhancing the generalizability of the findings. Integrating clinical and molecular data, as well as refining feature selection strategies, will further improve the discriminatory performance of radiomics models, particularly among malignant subtypes. Collectively, this work contributes to the establishment of a structured framework for CT-based radiomics in thoracic oncology and provides a foundation for its future translational ap-plication in personalized medicine.
